# Selective biomarkers for inflammation and infection are associated with post-operative complications following transperineal template prostate biopsy (TTPB): a single-centre observational clinical pilot-study

**DOI:** 10.1186/s40001-022-00807-8

**Published:** 2022-09-26

**Authors:** Nana Yaa Frempomaa Snyper, Joanne Pike, Kingsley Ekwueme, Iqbal Shergill, Stephen Fôn Hughes

**Affiliations:** 1 North Wales & Northwest Urological Research Centre, Betsi Cadwaladr University Health Board (BCUHB) Wrexham Maelor Hospital, Wrexham, Wales UK; 2 Maelor Academic Unit of Medical and Surgical Sciences, Betsi Cadwaladr University Health Board (BCUHB) Wrexham Maelor Hospital, Wrexham, Wales UK; 3 Faculty of Social and Life Sciences, Wrexham Glyndwr University, Wrexham, Wales UK; 4 Department of Urology, BCUHB Wrexham Maelor Hospital, Wrexham, Wales UK; 5 Department of Urology, BCUHB Glan Clwyd Hospital, Rhyl, Wales UK; 6 Pathology Division, 37 Military Hospital, Accra, Ghana

**Keywords:** Transperineal template prostate biopsy (TTPB), Benign prostatic hyperplasia (BPH), Prostate cancer (PCa), Cytokines, Urine beta-2 microglobulin, Post-operative complications

## Abstract

**Background:**

Prostate cancer (PCa) and benign prostatic hyperplasia (BPH) are the most common prostate disorders in the UK, which cause considerable ill health in older men. Transperineal template prostate biopsy (TTPB) has emerged as a reliable procedure for the histopathological diagnosis of PCa and BPH due to its higher cancer detection rates. Although antiseptic preparation and antibiotic prophylaxis are used to ensure safety in patients undergoing surgical intervention, post-operative complications, such as infection and bleeding are still unavoidable, resulting in re-admissions, with resource implications. Currently, there is no biomarker profile to predict outcomes or monitor patients during the post-operative course. The main aim of this single-centre observational clinical pilot-study was to investigate the role of inflammatory and infection biomarkers following TTPB and their association with post-operative complications.

**Methods:**

Forty-five patients scheduled for elective TTPB were recruited after informed consent at the Wrexham Maelor and Glan Clwyd Hospitals, North Wales, UK (*n* = 45). Prior to surgery, venous blood samples were collected at baseline and subsequently at 30, 120, and 240 min post-operatively. Urine samples were collected before and 120 min after the procedure. Serum procalcitonin (PCT), serum ferritin, and urine B_2_MG analysis were done using enzyme-linked fluorescent assay (ELFA) and the magnetic Luminex^®^ multiplex performance assay was used to analyse IL-6, IL-8, IL-10 and TNF-α plasma concentrations. Data on clinical outcomes were collected from patients’ medical records.

**Results:**

Following TTPB, significant (*p* ≤ 0.05) increases were observed in uB_2_MG, IL-6, IL-8, IL-10 and TNF-α. Significant decreases were observed in ferritin (*p* ≤ 0.05). No significant change was observed in PCT concentration (*p* ≥ 0.05). One patient developed an infection and severe haematuria post-operatively following TTPB.

**Conclusion:**

Although not confirmative, changes seen in biomarkers such as uB_2_MG, IL-10 and TNF-α in our observational clinical pilot-study may warrant further investigation, involving larger cohorts, to fully understand the role of these biomarkers and their potential association with post-operative complications such as infection and bleeding which can develop following TTPB for the diagnosis of PCa and BPH.

## Introduction

Prostate cancer (PCa) and benign prostatic hyperplasia (BPH) are the most common disorders of the prostate, which cause considerable ill health and have significant adverse effects on quality of life, especially in older men. In the United Kingdom (UK), about 52,254 new cases of PCa are diagnosed yearly (2016–2018), accounting for 27% of all new cancer cases in males [1]. BPH risk increases with age occurring in about 40% of men over 50 years.

Transperineal template prostate biopsy (TTPB) has emerged to be a well-tolerated procedure in the histological diagnosis of PCa and BPH. TTPB has a higher cancer detection rate and is highly recommended for repeat biopsy in men with previous negative biopsy, rising and persistently high PSA levels, and those with suspicious findings on multi-parametric magnetic resonance imaging (mpMRI) [2]. In addition, TTPB has become helpful in monitoring patients on active surveillance, as it offers a more accurate staging and guidance for subsequent management decisions [3, 4].

Although TTPB is well tolerated and strategies such as antiseptic skin preparation and antibiotic prophylaxis are used to ensure the safety of patients undergoing surgical procedures, the development of post-operative complications remains a serious concern [5]. There is still the potential for patients to develop complications such as infection, bleeding, and urinary retention due to the more extensive sampling of the prostate with multiple cores taken.

According to the British Association of Urological Surgeons (BAUS), following TTPB, (urinary tract infection) UTI occurs 1 in 100 patients (1%), sepsis occurs in less than 1 in 100 patients (< 1%), haematuria requiring emergency admission for treatment occurs in 1 in 100 patients (1%), clot retention 1 in 50 patients (2%) and acute retention of urine 1 in 20 patients (5%) [6].

Several studies have reported cases of UTI and sepsis following TTPB [7] [8]. In a recent evaluation of almost 500,000 prostate biopsies performed in the UK National Health Service from 2008 to 2019, TTPB was used in 98,588 cases. Observation after 28 days following TTPB indicated that 310 (0.31%) developed sepsis 757 (0.77%) developed an infection and 950 (0.96%) developed UTI [9]. Haematuria is a common manifestation of blood loss after TTPB [10, 11]. Urinary retention is by far the most common complication observed after TTPB with reported occurrence rates of 16% (*n* = 35) [10], 6.3% (*n* = 110) [12], and 7.9% (*n* = 379) [13]. Tamhankar et al*.* [9] identified acute urinary retention as the most common reason for non-elective hospital admission following TTPB over the past decade in the UK, resulting in 28.58% of admissions.

While some of these complications such as UTI and urosepsis risk are lower in TTPB (< 1.0%), it remains a well-known complication post-procedure. This may result in longer hospital stays, or re-admissions to hospitals with associated patient morbidity, resource implications, and reduced quality of life [10, 14].

TTPB, like any other surgical procedure, is often associated with a normal inflammatory response as part of the body’s normal healing process [15]. During TTPB, due to the insertion of needles into the prostate and consequent tissue removal from several sections of the prostate, the damage to the vascular endothelium following the procedure can be appreciated. Consequently, results in an inflammatory response and activation of the haemostatic system, which involves dynamic interactions with plasma, erythrocytes, platelets, leukocytes, endothelial cells, and cells of the tissue parenchyma leading to the expression and secretion of various biomarkers.

The inflammatory response involves the synthesis of acute-phase proteins such as C-reactive protein (CRP), procalcitonin (PCT) and ferritin, leukocyte attraction and transmigration through the endothelium, and accumulation at the site of injury [16, 17]. There is also the release of various pro- and anti-inflammatory cytokines, such as IL-6, IL-8, IL-10, and TNF-α, which also enhances the upregulation of adhesion molecules that facilitate leukocyte recruitment [18].

The actions of these biomarkers may collectively cause a substantial amount of damage to host tissue, prolong an inflammatory response following TTPB, and lead to post-operative complications. In case of complications, the tissue damage is more significant, resulting in increased serum levels of such biomarkers. These increases may be adequately sensitive to enable early diagnosis of the same complications.

C-reactive protein (CRP), coagulation tests, and patient’s history are routinely used to predict patients at high risk of post-operative infection complications [19, 20]. However, CRP is nonspecific, as its elevation is associated with various disorders, including surgery. Notably, there is also a sparse consensus on coagulation tests for predicting post-operative bleeding [21, 22].

Studies have reported that the concentrations of these PCT [23, 24], urine beta-2 microglobulin (uB_2_MG) [25, 26], serum ferritin [27–29] and cytokines (IL-6, IL-8, IL-10, and TNF-α.) [30–32] can be detected in serum in the early stages of bacterial infections and have been shown to be excellent predictors of early post-operative bleeding and infectious complications.

However, the single measurement of most biomarkers is inadequate and nonspecific, suggesting that a biomarker profile containing multiple markers could be more appropriate [33, 34].

Although the understanding of the inflammatory response following surgery has recently advanced, details of inflammation and infection biomarkers and their interactions in patients undergoing TTPB and post-operative responses such as infection and bleeding remain obscure. Also, further evaluation of these biomarkers during the post-operative period following TTPB will lead urologists to a well-supported consensus regarding improving post-operative care for better clinical outcomes.

Currently, no biomarker profiles are available to confidently forecast post-operative complications after surgical intervention of the prostate.

Clinically, developing biomarker profiles could help predict patients with or at increased risk of developing post-operative complications in real time. This could subsequently be used to identify patients who require intensified treatment or important changes to their care. Potentially, this could improve patient outcomes and health care provision, while also expanding existing knowledge in a field that is not well established.

The main aim of this single-centre observational clinical pilot-study was to investigate the role of inflammatory and infection biomarkers following transperineal template prostate biopsy (TTPB) and their association with post-operative complications.

## Methods

### Patient recruitment

Ethical approval for this study was received from the Welsh Research Ethics Service (REC) 4 committee (REC4: 12/WA/0117) and were carried out in accordance with the ethical rules of the Helsinki Declaration and Good Clinical Practice.

Forty-five (45) consecutive patients scheduled for elective TTPB at the Urology department of the Betsi Cadwaladr University Health Board (BCUHB) Wrexham Maelor Hospital and Glan Clwyd Hospital, North Wales, UK, was recruited after providing written informed consent (*n* = 45).

### TTPB procedure

TTPB was performed by Consultant Urological Surgeons as a day-case procedure with patients under either local (using 20 ml 1% xylocaine with adrenaline and 20 ml 1% lidocaine) or general (using 20 ml of 0.5% bupivacaine with adrenaline) anaesthesia in the lithotomy position. The procedure was performed as per standard protocol using a TRUS probe, brachytherapy template grid, and under direct ultrasound guidance, an 18-gauge biopsy needle was directed through the template grid to obtain biopsies. Biopsy cores were then taken from the left anterior, left lateral, left posterior, right anterior, right lateral, and right posterior zones of the prostate. The collected prostate biopsy samples were submitted for histopathological analysis at the Glan Clwyd Hospital.

### Blood samples

Venous blood samples were collected pre-operatively, then at 30 min, 120 min, and 240 min post-operatively. Urine samples were also collected pre-operatively and 120 min post-operatively. Aliquots of plasma, serum, and urine were prepared and stored at −80 °C at the Maelor Academic Unit of Medical and Surgical Sciences (MAUMSS) laboratory until analysis.

### Measurement of inflammatory and infection biomarkers

Serum PCT, serum ferritin, and urine B_2_MG concentrations in samples of patients was measured using VIDAS^®^ B.R.A.H.M.S PCT^™^ (PCT), VIDAS^®^ ferritin assay and VIDAS^®^ B2MG assay (Biomerieux, France), an automated quantitative test for the determination of analytes using an enzyme-linked fluorescent assay (ELFA). Plasma concentrations of IL6, IL8, IL10, and TNF-α were also determined performed using a customised Human High Sensitivity Cytokine Premixed Kit A supplied by R&D Systems, a Bio-techne brand, Minnesota, USA, using the Magnetic Luminex^®^ performance assay multiplex kit designed for analysis using the Bio-Rad^®^ Bio-Plex^®^ 100 (Bio-Rad Laboratories Inc, USA),

Details of complications (fever, pain, infection, bleeding, and urine flow rates) experienced by patients (*n* = 45) within 30 days post-biopsy were collected from patients’ medical records via patients’ notes and the BCUHB clinical portal. Significant complications were defined as any adverse event requiring intervention or readmission.

### Statistical analysis

Statistical analysis of data collected in this research was carried out using SPSS version 28 licensed. The Shapiro–Wilk test was used to check data distribution for normality, where *p* ≥ 0.05 was considered a normal distribution. When the data were normally distributed, the repeated measures analysis of variance (ANOVA) between samples test was used with a 5% level of significance. When ANOVA revealed significant variation (*p* ≤ 0.05), post hoc testing was performed using the Bonferroni test for pairwise comparisons of means. The Friedman test was used to analyse data that did not conform to normality (Shapiro–Wilk test *p* ≤ 0.05). Where the Friedman test yielded statistical significance (*p* ≤ 0.05), the Wilcoxon signed-rank test was used for subsequent tests.

When *p* ≤ 0.05, statistical significance was accepted. Parametric data were presented as mean and standard deviation as error bars, with non-parametric data reported as median and 10th percentiles.

## Results

Information provided in Table 1 represents a summary of the patient’s characteristics (e.g. age, height, weight, etc.).

**Table 1:** Summary of patients’ characteristics (*n* = 45)

Age	
Median	66 years
Range	54–77 years
Height	
Median	178 cm
Range	163–186 cm
Weight	
Median	86.9 kg
Range	60–138 kg
BMI	
Normal weight (18.5–24.9 kg/m^2^)	8 patients
Overweight (25.0–29.9 kg/m^2^)	23 patients
Obese class I (30.0–34.9 kg/m^2^)	7 patients
Obese class II (35.0–39.9 kg/m^2^)	5 patients
Obese class III (≥40.0 kg/m^2^)	2 patients

Information provided in Table 2 represents a summary of the surgical details.

**Table 2:** Summary of surgical details (*n* = 45)

Reason for TTPB	
Repeat biopsy due to negative TRUSPB	18 patients
Active surveillance	5 patients
Elevated PSA with abnormal findings on mpMRI	22 patients
PSA level before biopsy	
Median	6.8 ng/ml
Range	0.8–58.0 ng/ml
Prostate volume at biopsy	
Median	41 ml
Range	6.0–98.0 ml
Number of biopsy cores	
Median	29 cores
Range	7.0–61.0
Duration of TTPB	
Median	21 min
Range	5–45 min
Type of anaesthesia	
General	38 patients
Local	7 patients
Post-operative complications observed	
Infection (UTI/sepsis) and severe haematuria	1 patient
Severe pain	3 patients
Urine retention	1 patient
Complications requiring re-admission to hospital	1 patient

Information provided in Table 3 represents the histopathological characteristics of the study participants.

**Table 3:** Histopathological characteristics of study participants (*n* = 45)

		Number of cases	%
Diagnosis			
BPH		16	35.6
PCa		29	64.4
PCa Gleason Score and group grade			
Low grade	GS 3+3 = 6 (Group grade 1)	7	24.1
Intermediate grade	GS 3+4 = 7 (Group grade 2)	16	55.2
High grade	GS 4+3 = 7 (Group grade 3) GS 4+4 =8 (Group grade 4) GS 4+5 = 9 (Group grade 5)	6	20.7

### Changes in PCT concentrations following TTPB

**Fig. 1:**
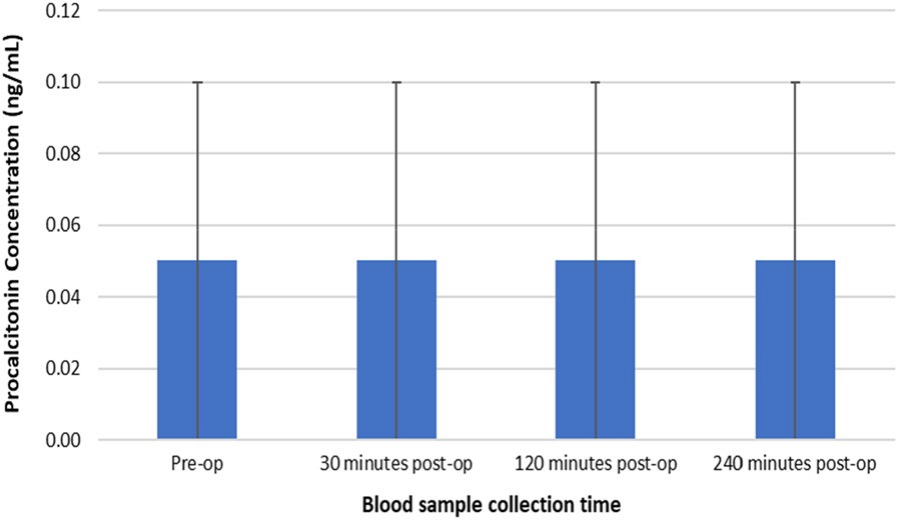
Changes in serum PCT concentrations following TTPB

Figure 1 represents changes in serum PCT concentrations following TTPB.

Friedman analysis showed no significant changes in serum PCT concentrations following TTPB, *X*^2^(3, *n* = 45) = 2.591, *p* = 0.459. Serum PCT concentrations remained unchanged from pre-operative levels (0.05 ± 0.05 ng/mL) to 30 min (0.05 ± 0.05 ng/mL), 120 min (0.05 ± 0.05 ng/mL) and 240 min (0.05 ± 0.05 ng/mL) post-TTPB.

Results presented as median, ± 10th percentile as error bars, *n* = 45.

### Changes in uB_2_MG concentrations following TTPB

**Fig. 2:**
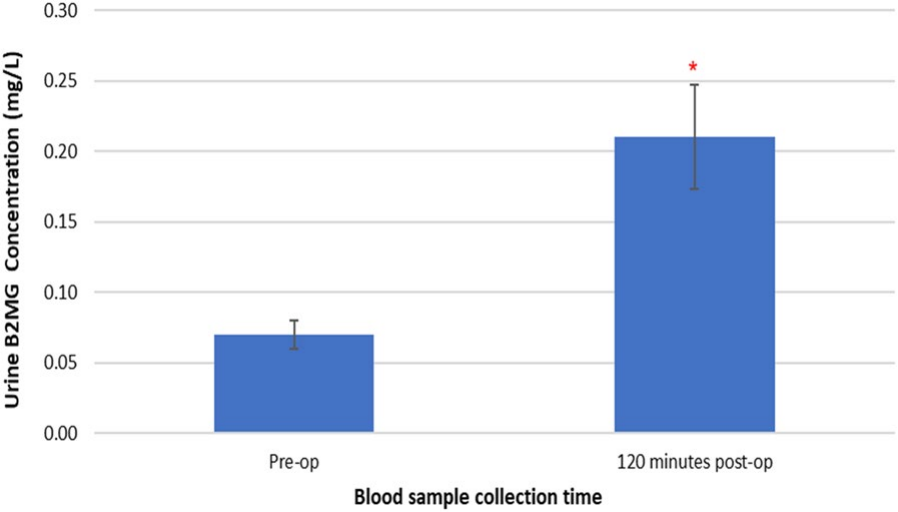
Changes in uB_2_MG concentrations following TTPB

Figure 2 represents changes in urine uB_2_MG concentrations following TTPB.

uB_2_MG concentrations increased from the pre-operative value (0.07 ± 0.01 mg/mL) to 0.21 ± 0.04 mg/mL at 120 min post-op. The change observed was found to be significant using the Friedman test, *X*^2^(1, *n* = 45) = 16.892, *p* < 0.001.

Results presented as median, ± 10th percentile, as error bars, statistical significance after post hoc analysis is represented when *p ≤ 0.05 compared with the pre-operative value, *n* = 45.

### Changes in serum ferritin concentrations following TTPB

**Fig. 3:**
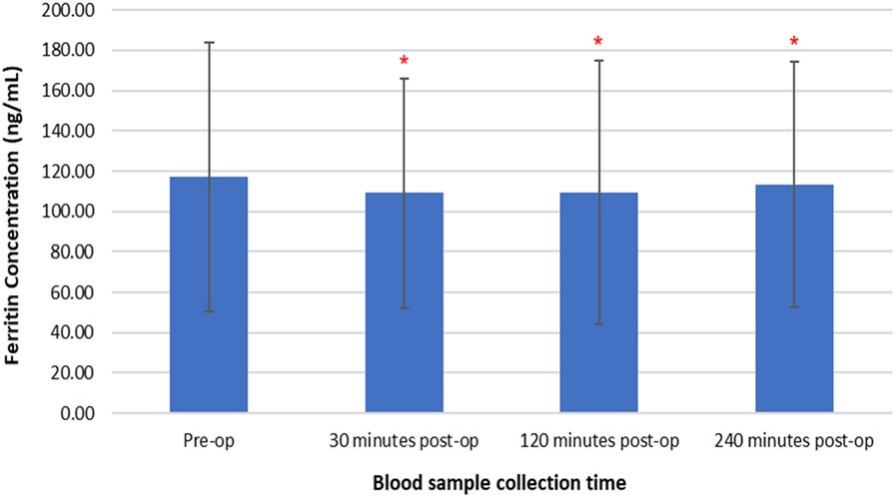
Changes in serum ferritin concentrations following TTPB

Figure 3 represents changes in serum ferritin concentrations following TTPB.

A Friedman test revealed that there was a significant decrease in serum ferritin concentrations following TTPB, X^2^(3, *n* = 45) = 39.411, *p* < 0.001. Pre-operative levels of serum ferritin, (117.1 ± 66.6 ng/mL), decreased at 30 min (109.3 ± 56.9 ng/mL), then a slight increase at 120 min (109.5 ± 65.3 ng/mL) and at 240 min post-op (113.5 ± 60.8 ng/mL) although recorded values were lower than pre-operative levels. Post hoc Wilcoxon signed-rank test showed that there was a significant decrease in serum ferritin levels at 30 min (*p* < 0.001), 120 min (*p* < 0.001) and 240 min (*p* = 0.008) post-TTPB, compared to pre-operative levels.

Results presented as median, ± 10th percentile, as error bars, statistical significance after post hoc analysis is represented when *p ≤ 0.05 compared with the pre-operative value, *n* = 45.

### Changes in plasma IL-6 concentrations following TTPB

**Fig. 4:**
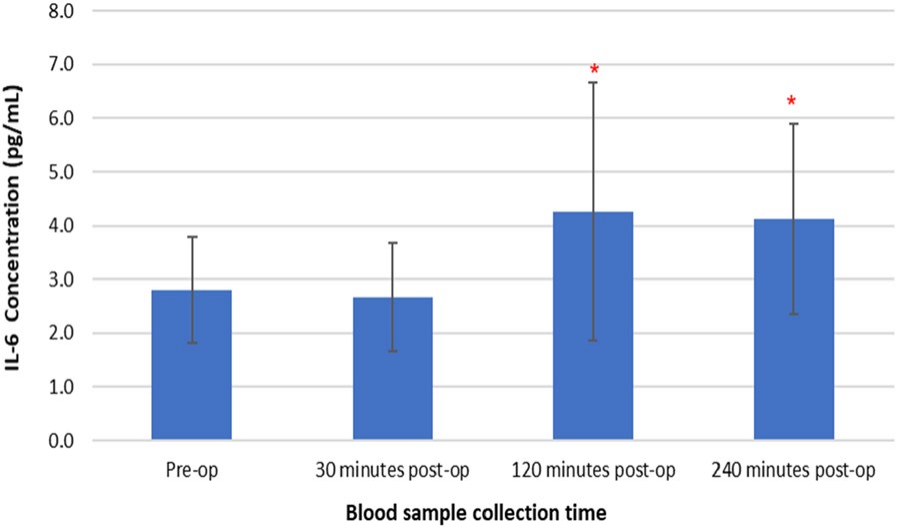
Changes in plasma IL-6 concentrations following TTPB

Figure 4 represents changes in plasma IL-6 concentrations following TTPB.

There was a significant increase in plasma IL-6 concentrations following TTPB as determined by the Friedman test *X*^2^(3, *n* = 45) = 35.054, *p* < 0.001. Pre-operative IL-6 values (2.8 ± 1.0 pg/mL) slightly decreased at 30 min (2.7 ± 1.0 pg/mL), then increased at 120 (4.3 ± 2.4 pg/mL), and 240 min post-op (4.1 ± 1.8 pg/mL). Post hoc Wilcoxon signed-rank test showed that there was a significant increase in plasma IL-6 concentrations at 120 min (*p* < 0.001) and 240 min (*p* < 0.001) post-TTPB, compared to pre-operative levels.

Results presented as median, ± 10th percentile, as error bars, statistical significance after post hoc analysis is represented when *p ≤ 0.05 compared with the pre-operative value, *n* = 45.

### Changes in plasma IL-8 concentrations following TTPB

**Fig. 5:**
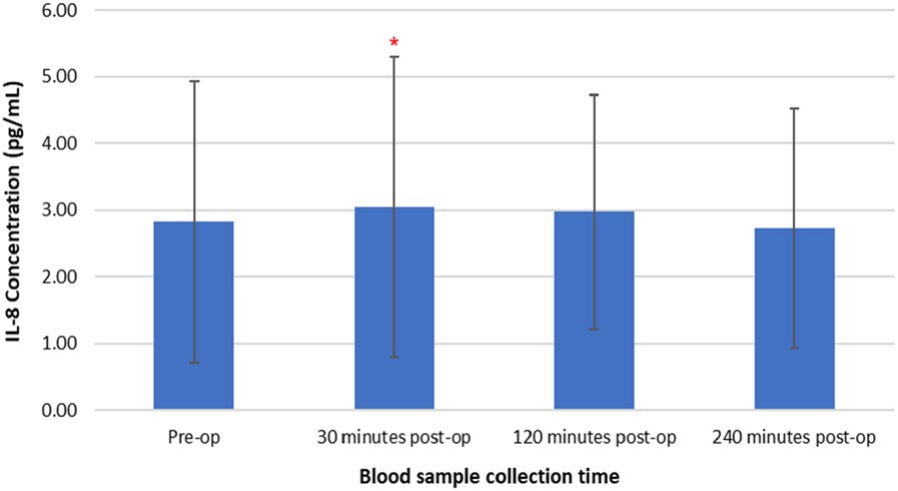
Changes in plasma IL-8 concentrations following TTPB

Figure 5 represents changes in plasma IL-8 concentrations following TTPB.

A Friedman test revealed a significant increase in plasma IL-8 concentrations following TTPB, *X*^2^(3, *n* = 45) = 10.324, *p* = 0.016). Baseline IL-8 increased from pre-operative (2.82 ± 2.12 pg/mL) to (3.05 ± 2.26 pg/mL) at 30 min, then a slight decreased at 120 (2.97 ± 1.75 pg/mL), and a further decreased at 240 min post-op (2.73 ± 1.80 pg/mL). Post hoc Wilcoxon signed-rank test showed that there was a significant increase in plasma IL-8 concentrations at 30 min post-TTPB (*p* = 0.030), compared to levels pre-operatively.

Results presented as median, ± 10th percentile, as error bars, statistical significance after post hoc analysis is represented when *p ≤ 0.05 compared with the pre-operative value, *n* = 45.

### Changes in plasma IL-10 concentrations following TTPB

**Fig. 6:**
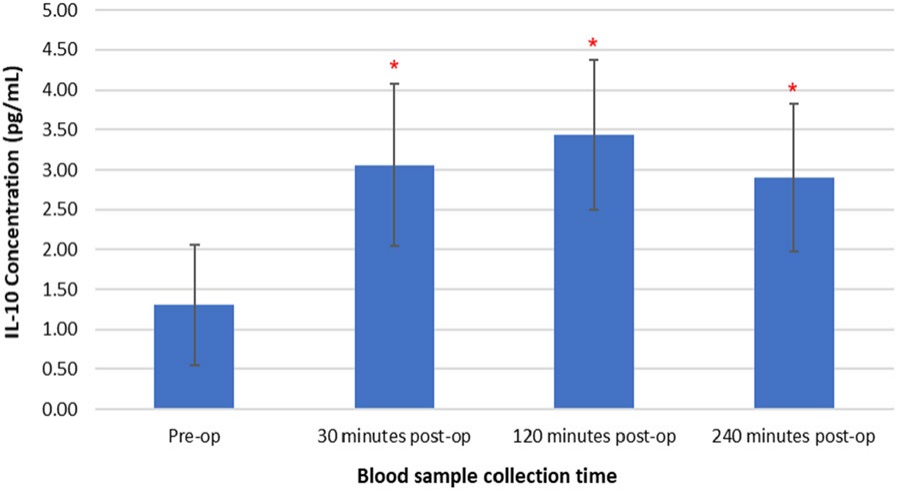
Changes in plasma IL-10 concentrations following TTPB

Figure 6 represents changes in plasma IL-10 concentrations following TTPB.

There was a significant increase in plasma IL-10 concentrations following TTPB as determined by the Friedman test, *X*^2^(3, *n* = 45) = 24.392, *p* < 0.001. Plasma IL-10 concentrations increased from the pre-operative value (1.30 ± 0.76 pg/mL) following TTPB to levels (3.06 ± 1.02 pg/mL) at 30 min, a further increase at 120 (3.43 ± 0.94 pg/mL), and then decreased at 240 min post-op (2.90 ± 0.92 pg/mL). Post hoc Wilcoxon signed-rank test showed that there was a significant increase in plasma IL-10 concentrations at 30 min (*p* < 0.001) and 120 min (*p* < 0.001) and a subsequent decrease at 240 min (*p* < 0.001) post-TTPB, when compared to pre-operative measurements.

Results presented as median, ± 10th percentile, as error bars, statistical significance after post hoc analysis is represented when *p ≤ 0.05 compared with the pre-operative value, *n* = 45.

### Changes in plasma TNF-α concentrations following TTPB

**Fig. 7:**
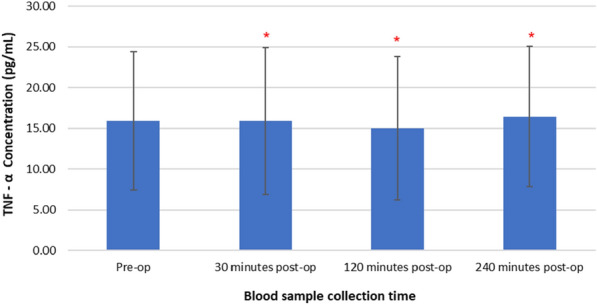
Changes in plasma TNF-α concentrations following TTPB

Figure 7 represents changes in plasma TNF-α concentrations following TTPB.

There was a significant increase in plasma TNF-α concentrations following TTPB as determined by the Friedman test, *X*^2^(3, *n* = 45) = 23.295, *p* = 0.022. Plasma TNF-α concentrations increased from the pre-operative value (15.89 ± 8.50 pg/mL) following TTPB to levels (15.91 ± 8.99 pg/mL) at 30 min, a decrease at 120 min (14.98 ± 8.80 pg/mL), and then increased at 240 min post-op (16.41 ± 8.62 pg/mL). Post hoc Wilcoxon signed-rank test showed a significant decrease in plasma TNF-α concentrations at 120 min (*p* < 0.001) and an increase at 30 min (*p* = 0.007) and 240 min (*p* = 0.003) post-TTPB when compared to pre-operative levels.

Results presented as median, ± 10th percentile, as error bars, statistical significance after post hoc analysis is represented when *p ≤ 0.05 compared with the pre-op value, *n* = 45.

### Changes in biomarkers of inflammation and infection and their association with post-operative complications

One participant (participant 11) with BPH, age 54 years, height 178.5 cm, weight 91 kg and BMI of 29 kg/m^2^ developed post-operative complications 3 days after TTPB. He was admitted to the emergency department of the Wrexham Maelor Hospital with pain, severe haematuria, and fever (38.5 °C). Routine blood results on readmission showed an increase in leukocytes—16.0 × 10ˆ9/L (normal reference range: 4.0—11.0 × 10ˆ9/L), neutrophils—13.8 × 10ˆ9/L (normal reference range: 1.7 −7.5 × 10ˆ9/L) and C-reactive protein—57 mg/L (normal reference range: < 5 mg/L). Urinalysis showed high leukocytes (3 +), protein (3 +), and blood (4 +) with visible blood clots. Further investigation revealed UTI leading to epididymo-orchitis, and he was hospitalised for 3 days and treated.

**Fig. 8:**
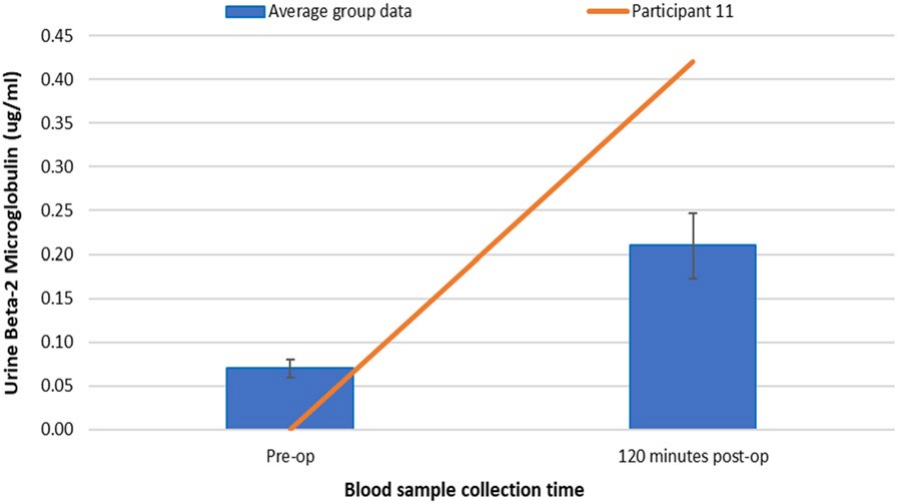
Changes in uB_2_MG concentrations for Participant 11 compared to the average group

Figure 8 shows the changes in urine uB_2_MG concentrations for participant 11 (pre-op—0.0 μg/ml and 120 min post-op—0.42 μg/ml) compared to the average study group who did not present any post-operative complications (pre-op—0.07 μg/ml and 120 min post-op—0.21 μg/ml).

Compared with the average group response, participant 11 had higher uB_2_MG concentrations post-TTPB. This could suggest that uB_2_MG could be a sensitive marker for predicting infection following TTPB.

Results presented as median, ± 10th percentile as error bars, *n* = 45.

**Fig. 9:**
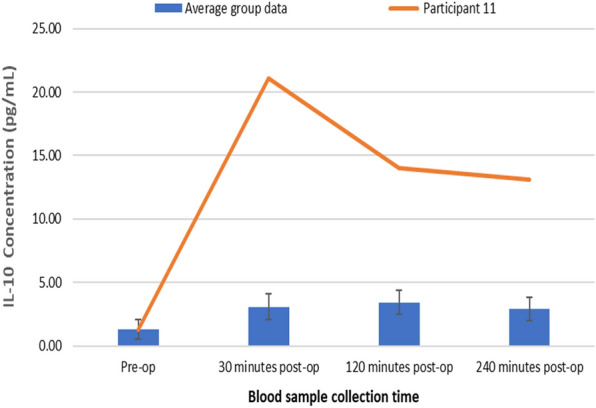
Changes in plasma IL-10 concentrations for Participant 11 compared to the average group

Figure 9 shows changes in plasma IL-10 concentrations for participant 11 (pre-op—1.29 pg/ml, 30 min post-op—21.1 pg/ml, 120 min post-op—14.05 pg/ml and 240 min post-op—13.09 pg/ml), compared to the average study group who did not experience any post-operative complications (pre-op—1.30 pg/ml, 30 min post-op—3.06 pg/ml, 120 min post-op—3.43 pg/ml and 240 min post-op—2.90 pg/ml).

Compared with the average group response, participant 11 had a high plasma IL-10 concentration at all time points post-TTPB. This further suggests IL-10 could be a sensitive biomarker for predicting infection and bleeding following TTPB.

Results presented as median, ± 10th percentile as error bars, *n* = 45.

**Fig. 10:**
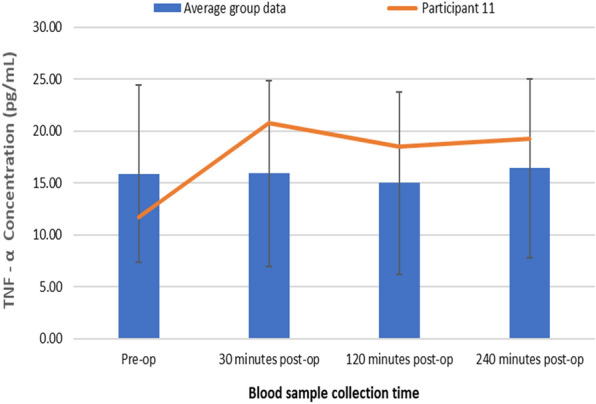
Changes in plasma TNF-α concentrations for Participant 11 compared to the average group

Figure 10 shows changes in plasma TNF-α concentrations for participant 11 (pre-op—11.72 pg/ml, 30 min post-op—20.73 pg/ml, 120 min post-op—18.49 pg/ml and 240 min post-op—19.26 pg/ml), compared to the average study group who present no post-operative complications (pre-op—15.89 pg/ml, 30 min post-op -15.91 pg/ml, 120 min post-op- 14.98 pg/ml and 240 min post-op—16.41 pg/ml).

Compared with the average group response, participant 11 had higher plasma TNF-α concentrations post-TTPB. This further highlights the potential of TNF-α as a sensitive biomarker for predicting UTI and haematuria following TTPB.

Results presented as median, ± 10th percentile as error bars, *n* = 45.

## Discussion

This single-centre observational clinical pilot-study intended to investigate the role of selective biomarkers for inflammation and infection (PCT, ferritin, uB_2_MG, IL-6, IL-8, IL-10 and TNF-α) following TTPB and their association with post-operative complications such as infection and bleeding. This study found that following TTPB, there are significant changes in some inflammatory and infection biomarkers, namely uB2MG, IL-6, IL-8, IL-10 and TNF-α.

PCT is a potent acute-phase reactant and increased PCT concentrations have been associated with sepsis and bacterial infection [23, 24]. Surprisingly, contrary to reported studies, the study found no significant changes in serum PCT concentrations following TTPB. Like the average group response, participant 11, who developed an infection following the procedure, showed no change in PCT concentrations. The observation of no significant change in PCT concentrations following TTPB could be due to the timings of sample collection (< 4 h). Probably the time was too short to observe an increase in PCT in patients with complications. PCT has a half-life of 24 h, concentrations begin to rise within 3 to 4 h of bacterial infection and reach their peak after 12–24 h [35]. This finding may also suggest that the post-operative course of PCT may differ based on the type of surgical procedure.

Several studies have reported that patients with UTI express increased concentration of uB_2_MG, and it has been suggested to be helpful in differential diagnosis and early detection of UTI [25, 26]. In agreement with previous reports [26, 27], our data follow a similar pattern of increased concentration following TTPB.

In agreement with Fang et al*.* and Jung et al*.* [25, 26], the patient (participant 11) who developed an infection following TTPB had higher uB_2_MG concentrations compared with the average group response (Fig. 8). This further suggests that uB_2_MG could be a sensitive marker for UTI following TTPB.

Increased serum ferritin concentrations have been associated with the severity of sepsis in children and the risk of early bacterial infection in adults [27–29]. In comparison to others [28–30], the present study demonstrated a significant decrease in serum ferritin levels at all times points measured post-TTBP, although these changes were minimal and were not of any clinical significance.

Increased IL-6, IL-8, IL-10 and TNF-α concentrations have been reported following surgical procedures and in the early stages of bacterial infections both in adults and children [30–32].

The present study found a significant increase in plasma IL-6 concentrations at 120 min and 240 min post-TTPB, which follows a similar pattern of change as reported after transurethral resection of the prostate (TURP) [36] and shock wave lithotripsy for kidney stones [37], suggesting an immune and stress (inflammatory) response to the procedure.

Plasma IL-8 concentrations showed a significant increase post-TTPB, and this supports the report by Yuan [36], who also observed an increase in IL-8 an hour post-TURP. The significant increase in plasma IL-8 concentrations observed in this study is consistent with reports that an acute-phase immune response occurs within 2 h of stimuli challenge [38].

Interestingly, Plasma IL-10 concentrations significantly increased following TTPB. Our data differ from Hughes et al*.* [37], who reported no significant changes to IL-10 concentrations post-operatively following shock wave lithotripsy (SWL), non-invasive urological procedure performed to treat small kidney stones. Normally, IL-10 is considered to have potent anti-inflammatory properties, but increased levels have been reported to be associated with infection. IL-10 inhibits pro-inflammatory cytokine response and killing of *Burkholderia pseudomallei* [39], and this pattern of increased IL-10 concentration was evident in the patient (participant 11) who developed complications in the present study.

The present study showed significant increases in plasma TNF-α concentrations following TTPB, with similar observations being reported by Hughes et al*.* [37] following SWL for the treatment of kidney stones. In line with reports Patel et al. [30] and Guan et al. [31], this study found that participant 11 had higher plasma TNF-α concentrations post-TTPB compared to the average group who did not develop any complications.

Collectively, the significant changes in uB_2_MG, plasma IL-10 and TNF-α concentrations following TTPB as reported by the patient (participant 11) who developed complications, suggest that these biomarkers could provide a sensitive and reliable tool for monitoring patients following TTPB. Thus, they could be considered as part of a biomarker profile to help identify those patients at increased risk of developing infection and bleeding complications post-operatively.

Apart from the relatively small number of patients (*n* = 45) included in this study, the current study has several limitations, in that; this study cannot exclude that there might have been some missed up or down-regulation of specific biomarkers during the later post-operative period. It would have been helpful to have a further sampling beyond 4 h, possibly 24–48 h after surgery. Additional sampling may accentuate any sustained changes to the various biomarkers and their association with complications. However, this was not feasible as TTPB is a strict day-case procedure at our hospital.

This study could not establish a significant association between biomarker changes and complications due to the low incidence of complications. In addition, it was not possible to draw clear conclusions about the specific effect of TTPB on biomarkers because of the lack of a control group of patients. It will also be interesting for future studies to assess the influence of antibiotic prophylaxis on the analysed biomarkers.

While this study might only initially be providing observational data, it can be appreciated that this research provides a scientific basis for future investigations involving more extensive multi-centre studies into routine and novel biomarkers and their correlation with clinical outcome. This would then subsequently provide further evidence for developing specific biomarker profiles that can be used in addition to, or in combination with, current management protocols for the identification of patients at higher risk of post-operative complications.

## Conclusions

Although not confirmative, changes seen to biomarkers such as uB_2_MG, IL-10 and TNF-α in our observational clinical pilot-study may warrant further investigation, involving larger cohorts, to fully understand the role of these biomarkers and their potential association with post-operative complications such as infection and bleeding which can develop following TTPB for the diagnosis of PCa and BPH.

## Data Availability

All data generated or analysed during this study are included in the published article.

## Abbreviations

BPH: Benign prostatic hyperplasia
BAUS: The British Association of Urological Surgeons
CRP: C-reactive protein
IL-1: Interleukin 1
IL-1β: Interleukin 1 beta
IL-6: Interleukin 6
IL-8: Interleukin 8
IL-10: Interleukin 10
NICE: National Institute for Health and Care Excellence
PCa: Prostate cancer
PCT: Procalcitonin
TTPB: Transperineal template prostate biopsy
TRUSPB: Transrectal ultrasound-guided prostate biopsy
TURP: Transurethral resection of the prostate
TNF-α: Tumour necrosis factor-alpha
UTI: Urinary tract infection
uB2MG: Urine Beta-2 microglobulin
